# Clinical outcomes of endoscopic mucosal resection for rectal neuroendocrine tumor

**DOI:** 10.1186/s12876-018-0806-y

**Published:** 2018-06-05

**Authors:** Jihye Kim, Jee Hyun Kim, Joo Young Lee, Jaeyoung Chun, Jong Pil Im, Joo Sung Kim

**Affiliations:** 1 Department of Internal Medicine and Liver Research Institute, Seoul National University College of Medicine, 101 Daehak-ro, Chongno-gu, Seoul, 03080 Republic of Korea; 2 Department of Internal Medicine, Seoul National University Boramae Hospital, Seoul National University College of Medicine, Seoul, 07061 Republic of Korea

**Keywords:** Neuroendocrine tumor, Rectum, Endoscopic mucosal resection, Efficacy, Prognosis

## Abstract

**Background:**

The incidence of rectal neuroendocrine tumors (NETs) is rapidly increasing because of the frequent use of endoscopic screening for colorectal cancers. However, the clinical outcomes of endoscopic resection for rectal NETs are still unclear. The aim of this study was to assess the rates of histologically complete resection (H-CR) and recurrence after endoscopic mucosal resection (EMR) for rectal NETs.

**Methods:**

A retrospective analysis was performed on patients who underwent EMR for rectal NETs between January 2002 and March 2015 at Seoul National University Hospital. Primary outcomes were H-CR and recurrence rates after endoscopic resection. H-CR was defined as the absence of tumor invasion in the lateral and deep margins of resected specimens.

**Results:**

Among 277 patients, 243 (88%) were treated with conventional EMR, 23 (8%) with EMR using a dual-channel endoscope, and 11 (4%) with EMR after precutting. The median tumor size was 4.96 mm (range, 1–22) in diameter, and 264 (95%) lesions were confined to the mucosa and submucosal layer. The en-bloc resection rate was 99% and all patients achieved endoscopically complete resection. The H-CR rates were 75, 74, and 73% for conventional EMR, EMR using a dual-channel endoscope, and EMR after precutting, respectively. Multivariate analysis showed that H-CR was associated with tumor size regardless of endoscopic treatment modalities (*p* = 0.023). Of the 277 patients, 183 (66%) underwent at least 1 endoscopic follow-up. Three (2%) of these 183 patients had tumor recurrence, which was diagnosed at a median of 62.5 months (range 19–98) after endoscopic resection. There was 1 case of disease-related death, which occurred 167 months after endoscopic treatment because of bone marrow failure that resulted from tumor metastasis.

**Conclusions:**

Although the en-bloc resection rate was 99% in rectal NETs, H-CR rates were 72–74% for various EMR procedures. H-CR may be associated with tumor size regardless of endoscopic treatment modalities.

## Background

Rectal neuroendocrine tumors (NETs) are uncommon tumors that account for approximately 10 to 17% of NETs [1]. The rectum is the third most common site of occurrence for NETs. Recently, the incidence of rectal NETs has rapidly increased worldwide, partly owing to rapid advances in screening endoscopy for colorectal cancer [2].

Several parameters have been suggested as predictive factors for malignant potential, including size, histological growth patterns, muscularis propria invasion, and lymphovascular invasion (LVI) [3]. At the time of diagnosis, approximately 80% of rectal NETs are smaller than 10 mm in diameter and do not show invasion or metastasis [4]. For small rectal NETs, the risk of metastasis is very low and local resection is thought to be curative [5, 6]. According to a previous study, metastasis occurs for less than 3% of tumors smaller than 10 mm in diameter, 5–15% of tumors 10–19 mm in diameter, and 80% of tumors at least 20 mm in diameter [7]. Endoscopic resection is widely accepted because of its minimal invasiveness, low cost, better quality of life after treatment, and patient tolerance. Rectal NETs that are smaller than 16 mm and do not show lymphatic or distant metastasis can be treated by local excision, such as endoscopic resection [8].

Conventional endoscopic mucosal resection (EMR) has been the endoscopic treatment of choice for rectal NETs. However, conventional EMR often produces incomplete resection of rectal NETs because even small rectal NETs can invade the submucosa. For this reason, various modified methods of EMR have been developed for the treatment of small rectal NETs, including EMR after precutting (EMR-P) and EMR using a dual-channel endoscope (EMR-D) [9–11]. Previous studies that assessed the prognosis of rectal NETs included surgically resected cases [6–8]. However, the literature has only included limited information on the efficacy of various EMR modalities and the prognosis of rectal NETs after EMR.

Therefore, we evaluated the clinical outcomes of EMR for rectal NETs in terms of the histologically complete resection (H-CR) rate and the recurrence rate after EMR. We further assessed the factors that were associated with H-CR.

## Methods

### Patients

We retrospectively reviewed the medical records of all patients who underwent EMR for rectal NET at Seoul National University Hospital (SNUH) (Seoul, South Korea) from January 2002 to March 2015. The inclusion criteria were as follows: (1) patients were at least 18 years old at endoscopic treatment, and (2) rectal NET had been pathologically diagnosed. The following patients were excluded from the study: (1) those who did not receive endoscopically complete resection, and (2) those who received endoscopic resection somewhere other than our center because we could not assess their clinicopathologic factors at initial treatment. In clinical practice, most patients who did not achieved endoscopically complete resection of rectal NETs receive immediate additional endoscopic or surgical treatment as salvage management. Therefore, we focused on the clinical outcomes of endoscopically resected rectal NETs without gross lesion.

Endoscopically complete resection was defined as resection of the lesion without grossly remnant tumor. Clinicopathologic and endoscopic data were retrospectively reviewed using the electronic medical records of our institution. The Institutional Review Board of SNUH approved this study (1605–052-760).

### Endoscopic procedures

All endoscopic procedures were performed by experienced endoscopists who are all gastroenterologist in SNUH. They have at least a few thousand colonoscopy experiences. The resection methods for rectal NETs included conventional EMR, EMR-P, and EMR-D.

We recommended endoscopic resection for NETs less than 10 mm in diameter without risk factors for recurrence and surgical resection for NETs larger than 20 mm or NETs with central erosion regardless of size. If the patients with rectal NETs refused surgery, they underwent endoscopic resection and were followed up with regular surveillance considering high risk of tumor recurrence. For rectal NETs of 10 to 20 mm in size, surgery or endoscopic treatment was chosen considering the risk of tumor metastasis and patient’s preference. In particular, surgery was strongly recommended as an initial treatment in rectal NETs, especially greater than 16 mm in size. There is currently no consensus regarding the optimal endoscopic treatment strategy among various EMR techniques [12–14]. The EMR method (conventional EMR, EMR-D, EMR-P) was selected according to endoscopist’s preference.

Conventional EMR and EMR-P were carried out with a single-channel colonoscope. EMR-D was carried out with a daul-channel colonoscope. Saline solution mixed with a small amount of indigo-carmine and diluted epinephrine (1:10,000) was injected into the submucosal layer beneath the tumor to reduce the risk of perforation and resection margin involvement. Subsequently, in all EMR modalities, snare resection was performed with an electrosurgical current. For EMR-D, the lesion was grasped by alligator forceps; snaring was then performed below the grasping forceps. For EMR-P, marking dots were made on the circumference of the lesion by electrocautery using a hook knife. Mucosal incision in EMR-P enables effective snaring without slippage of the snare.

### Evaluation of outcomes

The primary outcomes were H-CR and recurrence rates after endoscopic resection. Secondary outcomes were additive treatment for histologically incomplete resection (H-IR), procedure-related complications, and disease-related deaths.

All specimens were originally read by board-certified pathologists at SNUH between 2002 and 2015. Ancillary immunohistochemical (IHC) staining including D2–40, CD34, and CD31 in addition to hematoxylin and eosin (H&E) histologic examination was done to evaluate LVI in rectal NETs, if it is difficult or ambiguous to determine the presence of LVI in H & E staining. The tumor size, depth of invasion, resection margin status, differentiation, and presence of LVI were determined on the basis of the pathologic reports. H-CR was defined as being present when the lateral and deep margins of the specimens were free of tumor invasion. H-IR was considered to be present in all other cases. Maximum diameter was used as the measure for tumor size. Pathologic diagnosis was graded according to the 2010 World Health Organization (WHO) classification of tumors of the digestive system [15].

Local recurrence was defined as a pathologically confirmed diagnosis of NET at the same site more than 6 months after the initial treatment. If recurrence was diagnosed at the same site within 6 months after initial treatment, recurrence of rectal NET was considered as residual lesion rather than local recurrence.

Procedure-related complications were assessed, including bleeding and perforation. Procedure-related bleeding was classified as immediate when bleeding did not stop spontaneously and required intervention including argon plasma coagulation, electrocauterization, or hemoclips. Procedure-related bleeding was classified as delayed when bleeding occurred later than 24 h after endoscopic resection. Perforation was readily observed endoscopically or detected based on the presence of free air on a plain radiograph taken after the procedure.

### Follow-up

Subsequent treatments, including additional endoscopic resection or surgery, were recommended if H-IR or LVI were detected based on pathological results.

Patients who refused surgery received close observation with short-term endoscopic examination. Patients continued to undergo periodic follow-up with colonoscopy or sigmoidoscopy, and/or abdominal computed tomography (CT) scanning.

### Statistical analysis

The **χ** ^2^ or Fisher’s exact test was used to assess relationships among categorical variables, and the t test or Mann-Whitney U test was used for noncategorical variables. A multivariate logistic regression analysis was used to identify factors that were associated with H-CR. Variables with *p*-values less than 0.2 in the univariate analysis were considered for entry into the final multivariate analysis. The analysis results are presented as odds ratios (ORs) with 95% confidence intervals (CIs). Statistical analysis was performed using the Statistical Package for the Social Sciences software, version 15.0 for Windows (SPSS, Chicago, IL). Results were considered to be statistically significant for 2-sided *p*-values of less than 0.05.

## Results

### Patient selection

**Fig. 1:**
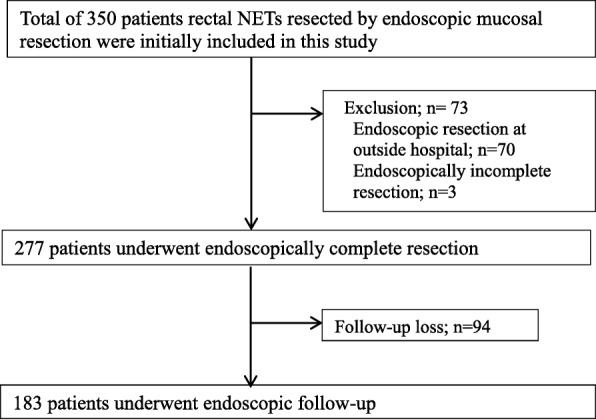
Flow chart showing the inclusion of study patients

A total of 350 patients who underwent EMR for rectal NETs were initially included in our study. However, the following 73 patients were excluded from our analysis: 3 patients who did not achieve endoscopically complete resection and 70 patients who had undergone endoscopic resection at an outside hospital. Thus, a total of 277 patients were included in the initial analyses of H-CR efficacy and factors contributing to H-CR for rectal NETs. Of these 277 patients, 183 (66.1%) underwent at least 1 endoscopic follow-up. Finally, these 183 patients were included in the assessment of long-term prognosis. A flow chart of the study inclusions and exclusions is shown in Fig. 1.

### Clinicopathologic characteristics

In the cohort of 277 patients, the median age at diagnosis was 50.9 years (range, 26–79) and 170 (61.4%) of the patients were male. The median distance from the anal verge was 7.1 cm (range, 1–16). Of the 277 patients, 243 (87.7%) were treated with conventional EMR, 23 (8.3%) with EMR-D, and 11 (4.0%) with EMR-P. Regarding the number of resection fragments, en-bloc resections were performed in 274 (98.9%) cases and piecemeal resections in the remaining 3 cases (1.1%).

The histologically estimated median tumor size was 4.96 mm (range, 1–22) in diameter. Rectal NETs less than 10 mm, 10–19 mm, and at least 20 mm in diameter were observed in 256 (92.4%), 20 (7.3%), and 1 (0.3%) patients, respectively. The histological assessments of resection margins indicated H-CR in 206 (74.4%) patients. LVI was found in 14 (5.1%) patients. According to the WHO classification**,** grade (Gr) 1 was found in 246 (88.8%) patients and Gr 2 in 3 (1.1%) patients The mitotic index was assessed in 211 (76.2%) patients, of whom only 9 (4.3%) were noted as having a high mitotic index (≥2/10 high-power field). Three (1.1%) patients had synchronous rectal NETs at initial diagnosis, and those lesions were removed at the initial endoscopic resection. Tumor invasion was confined to the submucosal layer in 269 (97.3%) patients.

Of the 277 patients, 183 (66.1%) continued to undergo follow-up colonoscopy or sigmoidoscopy. The median follow-up period was 32.1 months (range, 2.3–146.0). The clinicopathologic characteristics of the patients who underwent endoscopically complete resection for rectal NET are summarized in Table 1.

**Table 1:** The clinical and clinicopathologic characteristics of the patients who underwent endoscopically complete resection for rectal NETs

Variables	No.
Age, median (range), years	50.9 (26–79)
Gender, male	170 (61.4%)
Endoscopic appearance	
Polyp	22 (7.9%)
Submucosal tumor	255 (92.1%)
Distance from anal verge, median (range), cm	7.1 (1–16)
Treatment modalities	
Conventional EMR	243 (87.7%)
EMR-D	23 (8.3%)
EMR-P	11 (4.0%)
En-bloc resection	274 (98.9%)
Tumor size, median (range), mm	4.96 (1–22)
Group according to tumor size (%)	
< 10 mm	256 (92.4%)
10–19 mm	20 (7.3%)
≥20 mm	1 (0.3%)
Histologically complete resection	206(74.4%)
Negative lateral margin	240 (86.6%)
Negative deep margin	211 (76.1%)
Lymphovascular invasion	
Yes	14 (5.1%)
No	237 (85.5%)
Indeterminate	28 (9.4%)
Grade	
Gr1	246 (88.8%)
Gr2	3 (1.1%)
Not described	30 (10.8%)
Mitotic count	
< 2/10	202 (72.9%)
≥ 2/10	9 (3.2%)
Not described	66 (23.8%)
Presence of synchronous rectal NETs	3 (1.1%)
Tumor depth	
Limited to mucosa	5 (1.8%)
Submucosa	264 (95.3%)
Muscularis propra or deeper	0 (0%)
Indeterminate	8 (2.9%)
Follow-up	183 (66.1%)
Follow-up duration, median (range), months	32.1 (2.3–146.0)

Values are presented as median (range) or as numbers (%)

*Abbreviations*: *EMR* endoscopic mucosal resection, *EMR-D* EMR using a dual-channel endoscope, *EMR-P* endoscopic mucosal resection after precutting, *Gr* grade, *NET* neuroendocrine tumor

### Clinical outcomes

The overall rate of H-CR was 74.4%. A total of 3 patients underwent additive surgery at 1.1 months (range, 0.4–2.3) after initial endoscopic resection because the pathologic examination showed presence of NET cells at the resection margin (*n* = 2) and LVI (*n* = 1). Only 1 of the patient who underwent surgery after initial endoscopic resection had residual tumor on the surgical specimen. Based on endoscopic examination, this patient had not been suspected of having residual disease before surgery. No residual tumor was found in other 2 patients.

Complications occurred after endoscopic resection in 11 patients (4.0%). All complications were procedure-related bleeding, and 1 patient had delayed bleeding. All bleeding events were successfully managed by endoscopic clipping or coagulation therapy. There was no perforation after treatment.

### Factors associated with histologically complete resection

In our analysis of pathologic results, 206 (74.4%) patients were included in the H-CR group and 71 (25.6%) patients were included in the H-IR group. Table 2 shows factors associated with H-CR.

**Table 2:** Factors associated with histologically complete resection

Variables	H-CR (*n* = 206) (n, %)	H-IR (*n* = 71) (n, %)	*p* value
Age, median(range), years	50.0 (26–79)	52.7 (26–73)	0.33
Gender, male	123 (59.7%)	47 (66.2%)	0.333
Endoscopic appearance			0.489
Polyp	15 (7.3%)	7 (9.9%)	
Submucosal tumor	191 (92.7%)	64 (90.1%)	
Distance from anal verge, median, cm	6.3 (1–16)	6.0 (1–15)	0.852
Tumor size, median, mm	4.7 (1–22)	5.7 (2–12)	0.009
En-bloc resection	205 (99.5%)	69 (97.2%)	0.162
Lymphovascular invasion			0.215
Yes	10 (4.9%)	4 (5.6%)	
No	180 (87.4%)	57 (80.3%)	
Indeterminate	15 (7.7%)	10 (14.1%)	
Grade			0.668
Gr1	185 (89.8%)	61 (85.9%)	
Gr2	2 (1.0%)	1 (1.4%)	
Not described	19 (9.2%)	9 (12.7%)	
Mitotic count			N/A
< 1/10	148 (94.3%)	54 (100.0%)	
≥ 1/10	9 (5.7%)	0 (0.0%)	
Presence of synchronous rectal NETs	1 (0.5%)	2 (2.8%)	0.162

Values are presented as median (range) or as numbers (%)

*Abbreviations*: *H-CR* histologically complete resection, *H-IR* histologically incomplete resection, *N/A* not applicable, *NET* neuroendocrine tumor

There were no significant differences between the 2 groups in terms of age, gender, gross type of tumor, or distance from the anal verge. Further, there was no significant difference between the H-CR and H-IR groups in terms of procedure-related complications (7 [3.4%] for H-CR vs 4 [5.6%] for H-IR, *p* = 0.481) or recurrence (2 [1.0%] for H-CR vs 2 [2.8%] for H-IR, *p* = 0.272). The median tumor size was significantly smaller in the H-CR group than in the H-IR group (*p* = 0.009)*.*

The following variables had *p*-value less than 0.2 in the univariate analysis of associations with H-CR: tumor size, en-bloc resection, and presence of synchronous rectal NETs. However, when these variables were entered into a multivariate analysis, only tumor size remained significantly associated with H-CR (Table 3).

**Table 3:** Multivariate analysis to determine factors associated with histologically complete resection

	OR (95% CI)	*p* value
Tumor size	0.90 (0.82–0.99)	0.023
En-bloc resection	4.47 (0.39–51.5)	0.230
Presence of synchronous rectal NETs	5.17 (0.45–58.9)	0.186

*Abbreviations*: *CI* confidence intervals, *NET* neuroendocrine tumor, *OR* odds ratio

The H-CR rate according to endoscopic treatment modalities is shown in Table 4. The H-CR rate for rectal NETs in the conventional EMR group was 74.5%, which was similar to that in the EMR-D group (73.9%) and the EMR-P group (72.7%). There was no statistically significant difference in H-CR rates among the conventional EMR, EMR-D, and EMR-P groups (conventional EMR vs EMR-D, *p* = 0.114; conventional EMR vs EMR-P, *p* = 1.000; EMR-D vs EMR-P, *p* = 1.000).

**Table 4:** Histologically complete resection rates according to endoscopic treatment modalities

	H-CR rate, n (rate, %)	*p* value
Treatment modalities		0.99
Conventional EMR	181/243 (74.5%)	
EMR-D	17/23 (73.9%)	
EMR-P	8/11 (72.7%)	

*Abbreviations*: *H-CR* histologically complete resection, *EMR* endoscopic mucosal resection, *EMR-D* EMR using a dual-channel endoscope, *EMR-P* endoscopic mucosal resection after precutting

### Clinical outcomes during follow-up

During a median follow-up period of 32.1 months (range, 2.3–146.0), 183 (66.1%) patients were followed by endoscopic evaluation. Three (1.6%) patients developed tumor recurrence during the follow-up period, at a median of 62.5 months (range 18.5–98.0) after endoscopic resection.

Two of the 3 patients had local recurrence, while the remaining patient had distant metastasis to the liver and bone. All of these patients had tumors less than 10 mm in diameter confined to the submucosal layer and were treated with en-bloc resection: one patients underwent conventional EMR and 2 patients underwent EMR-D. In the pathologic examination, no patient had a high mitotic index. Among 2 patients who achieved H-CR at the initial endoscopic resection, one patient had no LVI and the other one had indeterminate LVI status. The 3 patients with recurrence received additive treatments: 2 underwent EMR and 1 underwent liver metastasectomy. One patient with residual lesion diagnosed at 2 months follow-up did not achieved H-CR and underwent additional electrocauterization as salvage treatment. All the patients with local recurrences and residual lesion remained free of disease during a median follow-up duration of 118.9 (range 60–167.3) months.

There was 1 disease-related death, which occurred 156 months after the endoscopic treatment because of bone marrow failure that resulted from tumor metastasis. The NET of this patient measured 5 mm in diameter without depression, ulceration, or a high mitotic index, and was assessed as endoscopically complete resection but not H-CR. Because the patient refused surgical resection, he underwent annual follow-up with sigmoidoscopy and abdominal ultrasonography. Seventy months after initial endoscopic treatment, metastasis in the liver was found on abdominal CT, while there was no evidence of local recurrence at sigmoidoscopy. The patient underwent liver metastasectomy. Surgical specimens from liver metastasectomy showed no evidence of LVI. However, the patient died 8 years after metasectomy because of disease progression in the liver and bone marrow.

## Discussion

It is well-known that rectal NETs have the smallest size of all gastrointestinal NETs [16]. Moreover, rectal NET was found to have the best prognosis of all NETs, with a 5-year survival rate of 88.2% [2]. For this reason, and because of the extremely low risk of lymph node metastasis, local excision is considered to be a sufficient treatment for lesions less than 10 mm in diameter [5]. However, there are currently no widely used guidelines regarding the management of rectal NETs 10–20 mm in diameter [17, 18].

Conventional EMRs cannot remove tumors with free vertical margins because of the presence of submucosal layer involvement, and increase the need for further treatment [19, 20]. As applied for deep resection of rectal NETs, endoscopic submucosal dissection (ESD) requires a long learning period, specific devices, and longer procedure times than conventional EMR. Therefore, several new endoscopic modalities have been developed, including EMR-D, EMR-P, and endoscopic submucosal resection with a ligation device (ESMR-L). These new modalities are reported to be effective for complete resection, as compared with ESD [21–23].

To date, several studies have reported factors that are associated with H-CR or prognosis in cases of rectal NET treated with endoscopic resection, including results for different treatment modalities [14, 21–24]. However, the studies have been limited by their small study cohorts. Previous study showed that treatment modality was the only factor that showed an independent association with the H-CR rate of small rectal NETs [24]. A meta-analysis indicated that ESD or modified EMRs (including EMR-D, EMR-L, EMR using a transparent cap, and ESMR-L) are superior to EMR, and that the efficacy of modified EMR nearly equals that of ESD in terms of the H-CR rate [14]. The efficacy of conventional EMR is known to be insufficient to remove rectal NETs by 64–82% of the H-CR rate [25–27]. According to previous studies, the H-CR rate of EMR-P and EMR-D were 87–100% and 74–83%, respectively [13, 25]. However, in the present study, we found that H-CR rates were 72–74% for various EMR procedures, and that only tumor size was significantly associated with the H-CR rate. We did not observe any significant differences in the H-CR rate among the conventional EMR, EMR-D, and EMR-P groups. Compared with the previous studies, the relatively high H-CR rate of conventional EMR and relatively low H-CR rate of modified EMR observed in this study might to be due to considerable proficiency in conventional EMR of our hospital. According to previous studies, the resection time is significantly longer for modified methods of endoscopic resection (including EMR-P, EMR-D, ESMR-L, and ESD) than for conventional EMR [23, 28]. If a physician has a relatively high H-CR rate of the conventional EMR due to lots of experience, the physician should be cautious about accepting a modified EMR unconditionally, considering that modified EMR techniques require longer procedure time and more equipment than conventional EMR.

In the present study, there were a total of 3 recurrences among the patients whose rectal NETs were completely removed through endoscopy, and who underwent at least 1 follow-up endoscopy. Two of the recurrences were local and 1 was a distant metastasis, with an overall median time to recurrence of 62.5 months. According to previous reports, the recurrence rate after endoscopic resection for rectal NET is approximately 0–4.2% [8, 14, 29, 30]. In a study of 304 patients with a median follow-up of 48 months, Park et al. reported 2 cases of recurrence after endoscopic resection for rectal NETs [31]. In a study of 153 lesions with a median follow-up of 31.0 months, Son et al. reported neither local recurrence nor metastasis to the regional lymph nodes or distal organs [24]. In a meta-analysis of 687 patients who rectal NETs were removed by endoscopic treatment, 6 patients had tumor recurrence during follow-up [14]. In our study, the recurrence rate after endoscopically complete resection was 1.6%, which is similar to the values that have been reported elsewhere [8, 14, 24, 31].

Of note, all cases of recurrence in our study involved a small tumor size (less than 10 mm in diameter), had a low mitotic index, and were confined to the submucosal layer. Our results differ from the recommendations of recent guidelines in terms of the development of tumor recurrence [32]. For example, follow-up is not recommended for rectal NETs less than 10 mm in diameter according to the National Comprehensive Cancer Network guidelines [32]. However, our study included 2 local recurrences, 1 residual lesion and 1 distant metastasis that led to death, all of which were observed among small rectal NETs (less than 10 mm) after endoscopic resection, and 1 of which even occurred after 8 years. Moreover, H-CR was also achieved in 2 of 3 recurrence cases. The possibility of an unexpected recurrence might be explained by the underestimated LVI state. Although a complete pathology reexamination using specific staining for LVI was not possible, 1 case of local recurrence with achieving H-CR and 1 case with residual lesion were confirmed not to accompany LVI by H&E staining with CD-34 staining. However, recently, the problem that LVI status in small rectum NET is not always accurately evaluated without special staining for LVI has been raised. D2–40 and CD-31 are the most useful specific marker for detect LVI in NETs [33]. According to recent study analyzed the LVI state in small rectal NETs using these IHC staining, LVI in small rectal NETs was observed in 46.7% of the cases [34]. However, recently published study in Korea, LVI rates in small rectal NETs were 25.0 and 27.9% in H&E and ancillary IHC staining, respectively. In this study, the concordance rate between 2 staining methods was 81.7% for detection of LVI, with strong agreement [33]. Even so, the low rate of LVI of rectal NETs observed in our study might be due to underestimated LVI assessment. When reviewed retrospectively, ancillary IHC staining, including D2–40, and CD31, was selectively performed in approximately 30% of patients in this study. The diagnosis of LVI during pathologic evaluation may be underestimated. In clinical practice, careful attention including confirmation using special staining for LVI should be paid to the interpretation of LVI.

All local recurrence cases in our study were treated by endoscopic resection, not by salvage operation. In principle, salvage operation should be recommended to patients with rectal NETs with unfavorable factors of recurrence such as H-IR, high mitotic index, or LVI. However, there has been no consensus on the appropriate salvage treatment for local recurrence of rectal NETs. In this study, 2 local recurrence cases were all small NET less than 10 mm and even in 1 case, there were no unfavorable factors of tumor recurrence. However, 1 of these 2 cases with local recurrence had indeterminate LVI state, and 1 case with residual lesion did not achieved H-CR. They also received endoscopic resection instead of surgery as salvage treatment. In real practice, many physicians facing indeterminate pathologic results choose additional endoscopic resection as salvage treatment of local recurrence, considering the preference of patients receiving endoscopic treatment, concerns about surgical risks, and the ease of access to endoscopy in South Korea. One residual lesion case with H-IR was also treated by endoscopic method. Gastroenterologists might not consider positive resection margin as a high risk factor for recurrence of tumor because of the cauterization effect of endoscopic resection.

A close relationship has been noted between tumor size and risk of metastasis [3, 7, 35]. Jetmore et al. noted that none of 56 patients with tumors less than 10 mm in size developed metastatic disease during a 32-year follow-up period [36]. However, Naunheim et al. raised concerns regarding the issue of size for NETs. They found that 13 (3.4%) of 388 patients with rectal NETs smaller than 10 mm in diameter had either metastatic disease on presentation or developed metastatic disease [37]. Although the risk of metastasis from small rectal NETs is generally low, some patients—even those with NETs smaller than 10 mm—may show metastasis [8]. Although small tumors generally have a benign course, post-excision recurrence developed for 4 (2.6%) of 152 tumors smaller than 10 mm in our study, which serves as a reminder that the behavior of these tumors is not easily predicted. Previous studies suggested that residual macroscopic disease is a poor prognostic factor [38]. However, in our study, 97.2% patients with H-IR were free of local recurrence and distant metastasis on follow-up, raising the question of whether H-CR is required to prevent disease progression. However, given the small number of cases with recurrence, further studies about the recurrence of rectal NETs after endoscopic resection are warranted to confirm this finding.

A recent study revealed that synchronous rectal NET at the initial diagnosis was associated with the development of development of metachronous rectal NETs [17]. Similar results were found in studies of colorectal adenoma. According to previous studies of metachronous colorectal adenoma after resection for colorectal cancer, synchronous colorectal adenoma is a risk factor for the development metachronous lesions [39, 40]. However, in our study, the presence of synchronous rectal NETs did not have a statistically significant association with NET recurrence after endoscopic resection. Nonetheless, our study included few cases of synchronous rectal NETs, which suggests that further investigation is needed with larger sample sizes.

There present study has several limitations. First, this was a single-center study, and the cases of rectal NETs in the modified EMR groups and the recurrence group were relatively small. For this reason, we could not analyze risk factors for recurrence after endoscopic resection. Although, we compared the efficacy of each EMR methods, there is a limitation that statistical power of this analysis is limited by numbers of patients in the modified EMR groups. Further studies should be needed to compare the efficacy among EMR techniques with a greater number of patients.

Second, because this study had a retrospective design, strategies for staging work-ups and surveillance were not standardized. The follow-up schedules may have differed across clinicians because there has not been any consensus regarding treatment strategies or surveillance for rectal NETs, in general. In addition, since the patients with rectal NETs were diagnosed over a long period of time in this study, a single criteria for the surveillance after initial treatment could not be applied to all patients. Accordingly, we decided to include patients who underwent at least 1 follow-up endoscopic evaluation in our analysis of clinical outcomes during follow-up. Third, the duration of follow-up, which is not long enough to assess recurrence of NETs, is limitation of our study. The median follow-up period of our study was 32.1 months (range, 2.3–146.0). In previous studies reporting clinical course after rectal NETs treatment, the median follow-up period ranged from 5 to 70 months. The follow-up period of our study is not comparatively short compared to the previous reports, however, the follow-up periods in our study varied widely, and some patients may have had relatively insufficient durations of follow-up. Considering the characteristics of slow growing rectal NETs and recurrence occurred after several years of initial treatment, a short follow-up period is a limitation of this study. Further study with longer follow-up periods should be needed to more accurately evaluate the recurrence risk of rectal NETs after endoscopic resection. However, the fact that several recurrences of rectal NETs after initial treatment were observed despite the relatively short follow-up period seems to be sufficient to raise awareness of monitoring for rectal NET recurrence. Fourth, this study did not include patients who underwent ESD and other modified EMR techniques such as ESMR-L and cap-assisted EMR (EMR-C). The aim of this study was to evaluate the efficacy of various EMRs on rectal NETs treatment, we excluded the patients with rectal NETs by resected by ESD. Because inter-endoscopist variation can have especially strong effects on clinical outcomes for ESD, which requires a high level of technical expertise as discussed previously [41]. Moreover, the inclusion period of this study was relatively long; had we extended the study to ESD, it may have required including early experiences with this modality. ESMR-L and EMR-C, which are known to be as convenient as conventional EMR, were not performed in our hospital due to the preference of the physicians. In particular, ESMR-L consistently showed a high H-CR rate of more than 90% in several studies. Therefore, further studies including various other EMR techniques such as ESMR-L and EMR-C are needed to confirm the efficacy of other modified EMR techniques not evaluated in this study [21, 42].

Despite these limitations, our study has important strengths: it is the largest single-center investigation of rectal NETs treated with EMR, and it includes a relatively long follow-up period with endoscopic examination. To the best of our knowledge, this is the first study of rectal NETs after EMR that has included analyses of the H-CR rate, factors associated with H-CR, and long-term prognosis after EMR.

## Conclusions

In conclusion, although the en-bloc resection rate was 99% for rectal NETs, H-CR rates were 72–74% for the investigated EMR procedures. H-CR may be associated with tumor size regardless of endoscopic treatment modalities. Further large-scale studies are needed to identify risk factors for recurrence after endoscopic resection, which will help to establish guidelines for the treatment of small rectal NETs.

## Abbreviations

CIs: Confidence intervals
CT: Computed tomography
EMR: Endoscopic mucosal resection
EMR-C: Cap-assisted EMR
EMR-D: Endoscopic mucosal resection using a dual-channel endoscope
EMR-P: Endoscopic mucosal resection after precutting
ESMR-L: Endoscopic submucosal resection with a ligation device
Gr: Grade
H&E: Hematoxylin and eosin
H-CR: Histologically complete resection
H-IR: Histologically incomplete resection
IHC: Immunohistochemical
LVI: Lymphovascular invasion
NET: Neuroendocrine tumor
ORs: Odds ratios
SNUH: Seoul National University Hospital
